# QTL mapping and transcriptome analysis of cowpea reveals candidate genes for root-knot nematode resistance

**DOI:** 10.1371/journal.pone.0189185

**Published:** 2018-01-04

**Authors:** Jansen Rodrigo Pereira Santos, Arsenio Daniel Ndeve, Bao-Lam Huynh, William Charles Matthews, Philip Alan Roberts

**Affiliations:** Department of Nematology, University of California, Riverside, California, United States of America; University of Missouri Columbia, UNITED STATES

## Abstract

Cowpea is one of the most important food and forage legumes in drier regions of the tropics and subtropics. However, cowpea yield worldwide is markedly below the known potential due to abiotic and biotic stresses, including parasitism by root-knot nematodes (*Meloidogyne* spp., RKN). Two resistance genes with dominant effect, *Rk* and *Rk*^*2*^, have been reported to provide resistance against RKN in cowpea. Despite their description and use in breeding for resistance to RKN and particularly genetic mapping of the *Rk* locus, the exact genes conferring resistance to RKN remain unknown. In the present work, QTL mapping using recombinant inbred line (RIL) population 524B x IT84S-2049 segregating for a newly mapped locus and analysis of the transcriptome changes in two cowpea near-isogenic lines (NIL) were used to identify candidate genes for *Rk* and the newly mapped locus. A major QTL, designated *QRk-vu9*.*1*, associated with resistance to *Meloidogyne javanica* reproduction, was detected and mapped on linkage group LG9 at position 13.37 cM using egg production data. Transcriptome analysis on resistant and susceptible NILs 3 and 9 days after inoculation revealed up-regulation of 109 and 98 genes and down-regulation of 110 and 89 genes, respectively, out of 19,922 unique genes mapped to the common bean reference genome. Among the differentially expressed genes, four and nine genes were found within the *QRk-vu9*.*1* and *QRk-vu11*.*1* QTL intervals, respectively. Six of these genes belong to the TIR-NBS-LRR family of resistance genes and three were upregulated at one or more time-points. Quantitative RT-PCR validated gene expression to be positively correlated with RNA-seq expression pattern for eight genes. Future functional analysis of these cowpea genes will enhance our understanding of *Rk*-mediated resistance and identify the specific gene responsible for the resistance.

## Introduction

Cowpea [*Vigna unguiculata* (L.) Walp] is a widely cultivated diploid legume species with 11 pairs of chromosomes and a haploid genome size of approximately 620 Mbp [1]. The species is native to Africa and is part of the warm-season legumes group, being closely related to common bean and soybean [2]. It is usually cultivated under resource-poor conditions in hot and dry environments, mainly in tropical areas of Africa, Asia, southern Europe, South America and southern USA [3]. Because of its high protein content in grain (25–30%), cowpea is a major source of protein and minerals for millions of people in sub-Saharan Africa and other developing countries [4]. Despite its comparatively strong adaptation to adverse environmental conditions as compared to other legume species such as common bean, cowpea is susceptible to many biotic stresses caused by insects, bacteria, viruses, fungi and nematodes, especially root-knot nematodes (RKN, *Meloidogyne* spp.) [5].

RKN are among the most important plant pests, causing annual crop losses accounting for billions of dollars globally [6]. They colonize plant roots and interfere with water and nutrients uptake by the root system, leading to poor development of the plant [7]. Several species of RKN infect cowpea, including *M*. *incognita* and *M*. *javanica* [8]. The use of resistant cultivars integrated into appropriate crop rotation schemes has been considered one of the most sustainable approaches for nematode management in cowpea.

Previous studies have reported that RKN resistance in cowpea is controlled by dominant genes [9–12]. Among these genes, a single dominant gene (*Rk*), effective against avirulent RKN isolates, was mapped on cowpea linkage group 11 (LG11) using genome-wide single nucleotide polymorphism (SNP) markers [13]. Another gene or locus (*Rk*^*2*^), which mediates a higher level of resistance than the one mediated by *Rk* alone and confers a broad-based resistance to different RKN populations has been described [11]. Despite the description of these two resistance genes and recent mapping of the *Rk* locus, the specific genes conferring resistance to RKN remain unknown.

In an effort to identify the genes underlying resistance response to RKN, we have mapped a resistance QTL localized on LG9 by analysis of a bi-parental recombinant inbred line (RIL) population, and used analysis of the transcriptome changes in two cowpea near-isogenic lines (NIL) to identify candidate genes for *Rk* and the newly mapped locus.

## Material and methods

### QTL mapping using a cowpea RIL population

A cowpea Recombinant Inbred Line (RIL) population of 84 F_10_ lines from the cross 524B x IT84S-2049, was used for quantitative trait loci (QTL) mapping. The breeding line 524B with *Rk*–type resistance was developed by the University of California, Riverside from a cross between California Blackeye No. 3 and California Blackeye No. 5. The breeding line IT84S-2049 was bred by the International Institute of Tropical Agriculture (IITA) and has a higher level of resistance than that conferred by the *Rk* gene alone and was determined to carry another resistance gene, *Rk*^*2*^, an additional dominant allele of *Rk* locus or a separate gene locus tightly linked to *Rk*. [11].

Cowpea plants were phenotyped for resistance reaction under controlled inoculations of *M*. *javanica* isolate 811 using seedling growth-pouches assay as described in Atamian et al. [14]. Briefly, cowpea seeds of each RIL and parental genotypes were grown in growth-pouches in a growth chamber with constant temperature of 27 ºC and 16 h of light per day. When adequate root systems were developed (about 15 days), each pouch was inoculated with approximately 1500 second-stage juveniles of *M*. *javanica*. Thirty five days after inoculation, each pouch was infused with egg mass dye solution (1 mg/L erioglaucine) and the egg masses on each root system were counted under a 10x-illuminated magnifier lens. The average number of egg masses per RIL was used to distinguish resistant from susceptible response. The experiment was arranged in a randomized complete block design with four replications. Each block included one plant of each RIL and 4 plants of each parental genotype which were used as controls.

Each RIL and parent was genotyped for 51,128 single nucleotide polymorphism (SNP) markers using the Cowpea iSelect Consortium Array (Illumina Inc., CA, USA). SNP calling was performed using GenomeStudio software (Illumina, Inc.) and SNP data were curated to eliminate monomorphic SNP loci, SNPs with > 20% missing and/or heterozygous calls, and segregation-distorted markers (MAF < 0.15). In addition, RILs with > 10% heterozygosity or those carrying many non-parental alleles were removed. The genetic map of the 524B x IT84S-20149 population was constructed using MSTmap [15] (http://mstmap.org/) at LOD 10 and the linkage groups (LGs) were numbered and oriented based on the cowpea consensus genetic map [16]. The following parameters were used to build the genetic map using the MSTmap software: Population type = RIL6; grouping LOD criteria = 10; no mapping size threshold = 2; no mapping distance threshold = 10 cM; try to detect genotyping errors = no; and genetic mapping function = Kosambi.

QTL mapping was performed using the Genome-wide efficient mixed model association algorithm (GEMMA) as described by Xu [17]. The significance of the detected QTL was acquired using the Bonferroni correction threshold (*P* = 0.05).

### Near-isogenic lines genotyping and QTL confirmation

Two sets of near-isogenic lines (NIL) (4 resistant and 4 susceptible) were genotyped using the cowpea iSelect platform with 51,128 SNPs to determine the presence and localization of the QTLs associated with resistance to *M*. *javanica* isolate “Project 811”. The resistant lines were derived from crossing the highly resistant RIL parent “IT84S-2049” (homozygous resistant, *Rk*^*2*^*Rk*^*2*^) to the recurrent parent California Blackeye 46 (CB46, homozygous resistant, *RkRk*) [18]. F1 plants from this cross were back-crossed to the recurrent parent CB46 to generate BC1F1, and then blind crosses were made between BC1F1 plants and CB46. Selfed seeds (BC1F2) from each BC1F1 plant were screened with *M*. *javanica* isolate “Project 811” in seedling growth-pouches for evaluation of resistance response based on egg-mass production, in comparison with the parents. Subsequently, crosses were made between heterozygous resistant BCF1 plants to produce BC1F2 which segregated for resistance, and resistant plants were selected for the next backcross cycle. Backcrossing was repeated for six cycles followed by single seed descent and phenotyping at the F6 was conducted to select for homozygous resistant lines. This approach was used to add the *Rk*^*2*^ gene into the CB46 background. In parallel, CB46 and a highly susceptible cultivar “Chinese Red” (homozygous susceptible, *rkrkrk*^*2*^*rk*^*2*^) were crossed as described by Huynh et al. [13]. Resistant CB46 was used as the recurrent parent in a backcrossing scheme to remove the *Rk* gene resistance to provide a susceptible CB46 (CB46 Null(5)). In brief, the recurrent parent CB46 (homozygous resistant, *RkRk*) was crossed with a highly susceptible cowpea landrace ‘Chinese Red’ (homozygous susceptible, *rkrk*), and the F1 was backcrossed to the recurrent parent CB46 to generate BC1F1. Blind crosses were then made between BC1F1 plants with CB46, and selfed seeds (BC1F2) of each BC1F1 plant were screened with *M*. *incognita* isolate Project 77 in growth pouches for variation in RKN resistance based on egg-mass production, in comparison with the parents CB46 and Chinese Red. Subsequently, crosses made from heterozygous resistant BCF1 plants (*Rkrk*), whose BC1F2 segregated for resistance, were selected for the next backcross cycle. Backcrossing was repeated for six cycles followed by single seed descent and phenotyping at the F6 to select for homozygous susceptible lines.

Two NILs, CB46 Null(5) (S) and CB46 72-1-3(6) (R), differing in the presence or absence of resistance alleles of both QTLs, were used to perform the RNA-seq assay.

### Characterization of QTL QRk-vu9.1 effect on RKN resistance

Four NILs were developed to characterize the QTL effect on resistance. To generate plants carrying only QTL *QRk-vu9*.*1*, one F2 and one BC1F1 population were created by crossing two NILs, CB46 Null(5) and CB46 72-1-3(6). The two populations were phenotyped for response to the aggressive *M*. *javanica* isolate 811 in growth-pouches and the numbers of egg masses per root system were counted as described before. Subsequently, 146 F2 lines, 21 BC1F1 lines, CB46 and the parents CB46 Null(5) and CB46 72-1-3(6) were genotyped using 15 SNP markers with the Kompetitive allele-specific polymerase chain reaction (KASP) assay (LGC Genomics Ltd., Hoddesdon, UK) [19]. Following marker trait association analysis to determine the effect of the QTL on nematode reproduction, the F2 lines were categorized into distinct phenotypic classes based on their response to egg mass production and SNP marker genotypes.

### Identification of candidate genes in BAC sequences

The cowpea BAC sequences (NCBI accessions AC279865-AC275219) [16] were used to determine the physical localization of candidate genes identified within the two QTL regions (*QRk-vu9*.*1*and *QRk-vu11*.*1*). To narrow down the BAC regions where the SNPs markers associated with the QTLs were contained, the online tool “HarvEST:web” (http://harvest-web.org) was used. Briefly, using target SNP markers a list of cowpea genes with common bean annotation was generated and filtered using as cutoff genes with hit length ≥ 350 bp and E-score ≤ 0.0001. The final list of candidate genes was obtained from filtering the data for genes present in chromosomes 4 and 9 of common bean, syntenic to cowpea LG11 and LG9, respectively. The genes localized in these QTLs were used for comparison to the RNA-Seq data.

### RNA-seq assay preparation

The same two NILs used for QTL validation, CB46 Null (S) and CB46 72-1-3 (R) were used to perform RNA sequencing. These two lines were inoculated with 3,000 freshly hatched second-stage juveniles of *M*. *incognita* (isolate Project 77) per plant in growth-pouches, as described before. The mock inoculation treatment consisted of 5 ml of deionized water. Root samples were collected at 3 and 9 days after inoculation and frozen until further processing.

For sample collection, root tissue from each treatment was excised using a sterile scalpel under a magnifying glass, immediately frozen in liquid nitrogen and stored at -80 ºC. Similarly, root tissue was collected from equivalent root regions of the control plants (tissue near root tips of secondary and tertiary roots). Galled tissue was excised by cutting the region immediately adjacent to the root-gall to minimize the amount of non-infected tissue included in the assays. Each sample consisted of galls pooled from 10 plants. Extra roots were stained with acid fuchsin to evaluate nematode root penetration and migration as described by Byrd et al. [20].

Total RNA was extracted using Spectrum Plant Total RNA kit (Sigma-Aldrich Corp., St. Louis, Mo., USA) according the manufacturer’s protocol. RNA was treated with RNase-Free DNase set (QIAGEN Inc., Valencia, CA, USA) and purified using RNeasy MinElute Cleanup kit (QIAGEN Inc., Valencia, CA, USA). Quality and concentration of RNA samples were determined using Agilent 2100 Bioanalyzer (Agilent Technologies, Santa Clara, CA, USA). Four multiplexed libraries for next generation sequencing were prepared using NEBNext Ultra RNA library prep kit for Illumina (New England BioLabs Inc., Ipswich, MA, USA) according to the manufacturer’s instructions. Paired-end sequencing was performed by Illumina HiSeq 2500, from which single-end (1 x 100 bp) sequence data were used for analysis. Raw sequence data from this study were deposited at NCBI Sequence Read Archive (SRA) as bioproject accession PRJNA387626. RNA-seq data were produced by the Institute for Integrative Genome Biology (Genomics Core Facility, University of California–Riverside).

### Analysis of differentially expressed genes

The raw data were analyzed by the Institute for Integrative Genome Biology (Bioinformatics Core Facility, University of California–Riverside). Briefly, the raw reads were cleaned by removing adaptor sequences, empty reads, low-quality sequences, and short reads. The high-quality reads were aligned to the *Phaseolus vulgaris* genome (V1.0), available in Phytozome (Joint Genome Institute), using Tophat2/Bowtie2 [21, 22]. Reads overlapping with the annotation range of interest were counted for each sample using the “summarizeOverlaps” function [23]. Read counting was performed for exonic gene regions in a non-strand specific manner. The analysis of differentially expressed genes was performed by edgeR package [24] from Bioconductor. The log_2_ fold change (log_2_FC) of expression profiles (as RPKM) was computed between the inoculated and mock inoculated plants (control); and resistant and susceptible NILs (control) for each treatment. A false discovery rate (FDR) ≤ 0.1 and log_2_FC ≥ ±1 was used as the threshold to judge the significance of each gene expression difference. GO analysis was performed using a web-based tool agriGO (http://bioinfo.cau.edu.cn/agriGO) [25], based on available *Phaseolus vulgaris* annotations. The differentially expressed genes (DEG) were compared with candidate genes found in the QTL regions.

### Validation of RNA-Seq data by qRT-PCR

Quantitative reverse transcription PCR (qRT-PCR) analysis was performed to validate the RNA-Seq results for eight gene transcripts whose expression differed by more than 1.0-fold (up or down-regulated) between the CB46 72-1-3 and CB46 Null NILs after inoculation. Gene-specific primers for qRT-PCR were designed using PrimerQuest Tool (https://www.idtdna.com/-Primerquest/Home/Index), OligoAnalyser 3.1 (https://www.idtdna.com/calc/analyzer) and UNAFold (https://www.idtdna.com/unafold/Home/Index) tools. cDNA was synthesized using SuperScript III First-Strand Synthesis System for RT-PCR (Cat. 180080–051, Invitrogen, Carlsbad, CA, USA) and real-time quantification was performed using a Bio-Rad MyIQ5 system (Bio-Rad, Hercules, CA, USA) and the Bio-Rad iQ SYBR Green Supermix Kit (Cat. 170–8882). An Elongation Factor (EF) gene from cowpea, based on *Phaseolus vulgaris* annotation, was used as a stably expressed internal reference. qRT-PCR was performed using three technical replicates for each sample from three biological replicates using a 15 μl reaction volume. The conditions for amplification were as follows: 3 min denaturation at 95°C, followed by 40 cycles of 95°C for 10s, 62°C for 30s, and 72°C for 30s. Relative gene expression was calculated using the 2^-ΔΔCt^ method [26].

### Characterization of candidate genes based on *V*. *unguiculata* gene expression atlas

For a better characterization of the best three candidate genes, the complete sequences of each gene were aligned to a cowpea transcriptome data bank, *Vigna unguiculata* Gene Expression Atlas (VuGEA), available at http://vugea.noble.org/index.php [27]. The expression profiles in different tissues, putative annotation, functional annotation and categorization of the two best hits and their co-expressed transcripts for each sequence were used to characterize the candidate genes, when this information was available. The first two hits shown were also used to perform a blastx (https://blast.ncbi.nlm.nih.gov/Blast.cgi/) to compare with other genes.

### Protein domain characterization from candidate genes

To characterize the proteins encoded by the best candidate genes, the complete sequence of each gene was used to predict the full length of amino acids. The similarity between the genes selected and their homologs in *Phaseolus vulgaris* was used to find the exons by comparison. Once the intronic region was removed the sequences were translated to amino acids using the Expasy translate tool (http://web.expasy.org/translate/) [28]. Because the three genes selected are homologous to three TIR-NBS-LRR genes in *Phaseolus vulgaris*, the cowpea proteins were aligned and the percentage of identity between them determined using the online tool Clustal Omega from EMBL-EBI bioinformatics web service [29], available in (http://www.ebi.ac.uk/Tools/msa/clustalo/). The conserved domain for each protein was found using the InterProScan [30], and the Prosite–MyDomains—Image creater (http://prosite.expasy.org/mydomains/) was used to draw the protein domains. Protein sequences were used to query the cellular localization using CELLO2GO: A Web Server for Protein subCELlular LOcalization Prediction with Functional Gene Ontology Annotation (http://cello.life.nctu.edu.tw/cello2go/) [31]. Each protein predicted from the candidate genes was used to search for other proteins sharing amino acid similarity with them. For that, a Blastp (NCBI, https://blast.ncbi.nlm.nih.gov/Blast.cgi) was performed with each predicted protein and the first 100 proteins found were aligned to the predicted cowpea protein and a phylogenetic tree was built with MEGA 7 [32]. The distances were inferred using the Neighbor-Joining method [33] and the Poisson correction method [34]. An additional phylogenetic tree was generated using the complete sequence of amino acids of three predicted cowpea proteins, 11 proteins sharing similarity to each one and a *Prunus persica* protein (outgroup).

## Results

### Quantitative trait loci mapping using a cowpea RIL population

**Fig 1:**
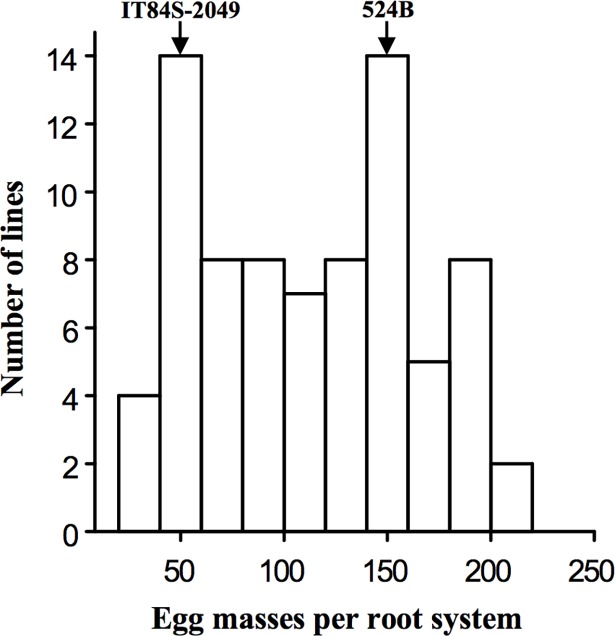
Variation in RKN resistance traits within the 524B x IT84S-2049 RIL population. The number of RKN egg masses per root system was measured in pouch tests conducted in a growth chamber.

In the phenotype assays of the 524B x IT84S-2049 RIL population, the mean number of *M*. *javanica* egg masses per root system (EM) varied among the RILs, ranging from 31 to 216 EM (Fig 1). The resistant parent IT84S-2049 supported fewer EM (48) than the susceptible parent 524B (153) and the segregation in the RIL population followed a bi-modal distribution.

A linkage map was generated based on the previously published iSelect genotyping assays [16] and utilized for QTL detection. The resulting genetic map contained 14,202 SNP markers on 933 unique map positions distributed over 11 LGs, covering a total genetic distance of 909 cM with an average marker distance of 1.0 cM (S1 Fig).

**Fig 2:**
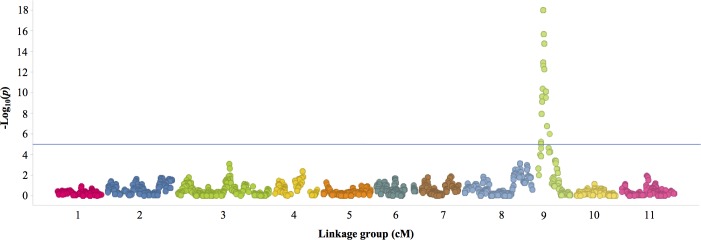
Manhattan plot of SNPs associated to *M*. *javanica* resistance. The blue line represents the Bonferroni correction using a threshold of *p* = 0.05. The Infinite (INF) values of -log_10_(p) were represented as a numeral “18”.

One major QTL associated with resistance to EM production by *M*. *javanica* was detected in the 524B x IT84S-2049 RIL population (Fig 2). This QTL, named *QRk-vu9*.*1*, which explained approximately 64% of total phenotypic variance (PVE) was mapped on linkage group LG9 at position 13.37 cM, flanked by SNP markers 2_14948 and 2_38272 (-log_10_p = 4.21 –INF) and spanned 13471 cM (from 4.277–17.748 cM). Favorable (low EM) alleles for resistance within *QRk-vu9*.*1* were contributed by the resistant parent IT84S-2049.

### NIL genotyping and QTL confirmation

Two sets of CB46 NILs with contrasting EM phenotypes (4 resistant and 4 susceptible) were genotyped using 51,128 SNP markers. A region of approximately 8.5 cM was identified on linkage group 9 based on the 524B x IT84S-2049 genetic linkage map containing polymorphisms that distinguished the two phenotypes (S1 File). Resistant NILs were homozygous for the donor (IT84S-2049) alleles within the QTL region associated with resistance and, as expected, the susceptible NILs were homozygous for the recurrent parent (CB46) alleles. This RKN resistance region from the donor contained the SNP markers that also flanked the QTL *QRk-vu9*.*1* mapped on linkage group 9 of the 524B x IT84S-2049 RIL population. The susceptible lines were mostly homozygous for the CB46 background except for a common region of approximately 50 cM on LG11 that was homozygous for the susceptible donor (Chinese Red) alleles. This region contained SNP markers that also flanked the major QTL *QRk-vu11*.*1* previously mapped on linkage group 11 [13] (S2 File).

Two of the NILs (CB46 Null(5), S; CB46 72-1-3(6), R), 98% identical with a total of 966 polymorphic SNPs, were used to perform the RNA-seq assay. Only six heterozygous SNP markers were found in the resistant line and seven in the susceptible line, which confirmed their high level of homozygosity. The two percent difference can be explained by the presence and absence of alleles of both QTLs mapped between the NILs (S3 File).

### Characterization of QTL QRk-vu9.1 effect on RKN resistance

**Fig 3:**
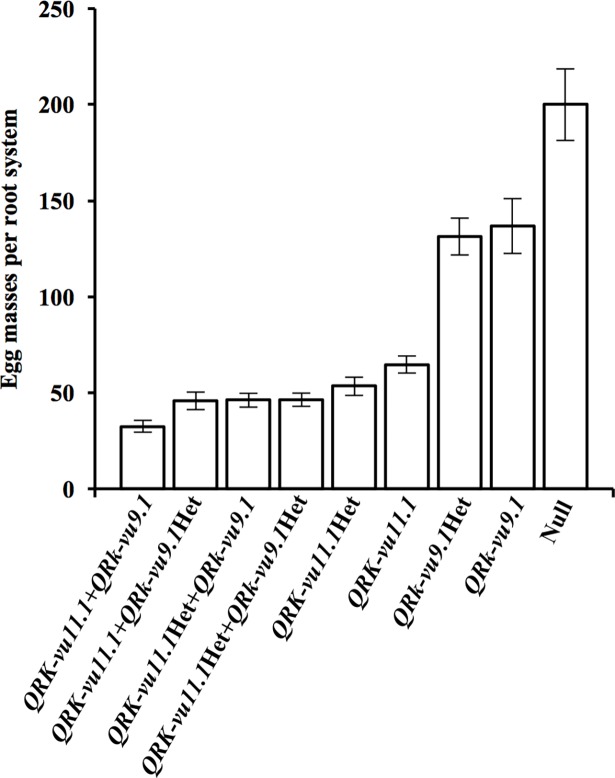
Mean numbers of *Meloidogyne javanica* egg masses per root system in nine groups of near-isogenic lines (NIL) based on allele condition of two QTLs. The mean number of egg masses per root system was measured in growth-pouch inoculation assays conducted in a controlled environment chamber. * Number of NILs per group; Het = heterozygous.

To characterize the effect of *QRk-vu9*.*1* on RKN resistance, a F2 population was developed by crossing the two NILs (described above) which had 966 polymorphic SNPs. From the 160 lines phenotyped for *M*. *javanica* EM production, 146 were genotyped with 15 polymorphic SNP markers. After genotyping, 81 F2 lines were clearly distinguished by the presence or absence of both QTLs and were grouped into nine clusters. The average EM of the 81 lines and the 35 NILs (Fig 3) revealed the high resistance to EM production of NILs with both QTLs in the homozygous resistant condition, followed by those NILs carrying both QTLs with markers heterozygous for either or both QTLs. NILs carrying only *QRk-vu11*.*1* (homozygous resistant) exhibited an intermediate resistance response against *M*. *javanica*, which was weaker compared to the lines carrying both QTLs in the homozygous condition (*P*<0.05). Lines carrying only *QRk-vu9*.*1* in the homozygous resistant condition showed weak resistance to *M*. *javanica* EM production. This weak resistance phenotype, when combined with positive alleles from *QRk-vu11*.*1* resulted in enhanced resistance against *M*. *javanica*. This weak effect, when combined with positive alleles from *QRk-vu11*.*1* resulted in a much higher level of resistance.

### Candidate genes under QTL regions

The SNPs within each QTL interval were used to identify BAC sequences available from Munõz-Amatriain et al. [16] underlying the QTL regions. The genes identified in each QTL region with homology to common bean (*Phaseolus vulgaris*) were listed and filtered based on the best synteny between the two crop species. Under the *QRk-vu9*.*1* region, 103 genes were identified with strong hits after filtering. Genes encoding TIR-NBS-LRR (1 gene), LRR protein kinase (2 genes), glycine-rich protein (1 gene), pathogenesis-related thaumatin superfamily protein (1 gene), MAP kinase 7 (1 gene) and protein kinase superfamily protein (2 genes) were considered as potential candidates for genes conferring resistance to root-knot nematodes. Under the *QRk-vu11*.*1* region, 165 genes were found in the BAC sequences, including NB-ARC domain-containing disease resistance protein (45 genes), LRR protein kinase (2 genes), TIR-NBS-LRR (11 genes), LRR-NB-ARC domain-containing disease resistance protein (3 genes) and disease resistance family protein/LRR family protein (3 genes). The complete list of genes found in both QTL regions in the BAC sequences is given in S4 File.

### Nematode root penetration and migration

**Fig 4:**
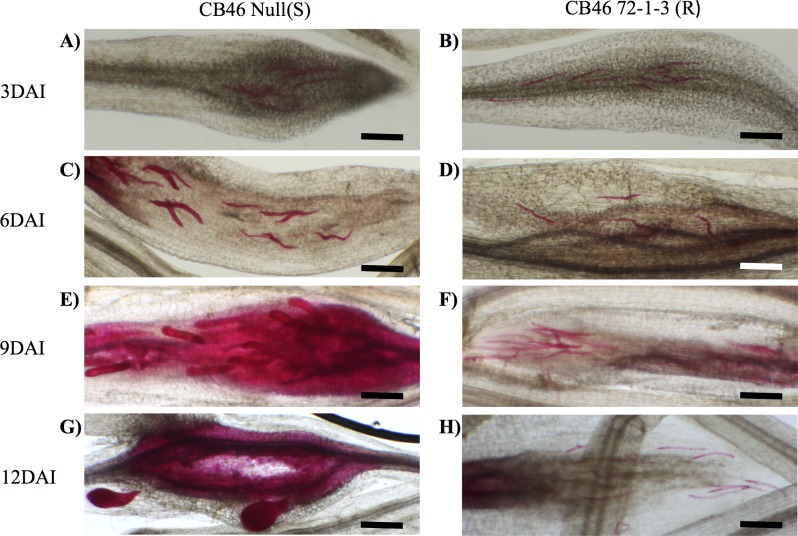
Nematode penetration and development on resistant and susceptible cowpea NILs. Roots of 12-day-old seedlings were infected with an equal number of RKN juveniles, and stained with acid fuchsin at different DAI. (A) NIL-S, 3 DAI. (B) NIL-S, 3 DAI. (C) NIL-S, 6 DAI. (D) NIL-S, 6 DAI. (E) NIL-S, 9 DAI. (F) NIL-S, 9 DAI. (G) NIL-S, 12 DAI. (H) NIL-S, 12 DAI. Bars = 250μm.

To visualize nematode root penetration and the effects of resistance response on RKN development, the CB46 Null (S) and CB46 72-1-3 (R) NILs were infected with freshly hatched J2s *of M*. *incognita* (isolate Project 77) and stained with acid fuchsin for nematode visualization at different days after inoculation (3,6,9,12 DAI). Similar numbers of nematodes were observed in both NILs at 3 DAI (Fig 4A and 4B), indicating that the resistance controlled by *QRk-vu11*.*1/ QRk-vu9*.*1* loci had no effect on nematode penetration and migration of nematodes in cowpea roots and is most likely dependent on effector recognition by plant proteins during the first steps of feeding site establishment. Differences in the development of nematodes were observed starting at 6 DAI. A clear distinction of juvenile development was observed among the two genotypes (Fig 4C and 4D). A similar trend was observed at 9 DAI, when developed juveniles were observed in susceptible lines (Fig 4E) but not in resistant lines, in which J2 remained undeveloped (Fig 4F). In agreement with the effect of resistance on nematode development in resistant plants, at 12 DAI young females were visible in susceptible plants (Fig 4G), while only undeveloped J2 were observed in the resistant plants (Fig 4H).

### RNA-seq analysis

The Illumina platform produced a total of 541,386,496 reads of an average 100 bp in length from 16 cDNA libraries (2 NILs x 2 time-points x 2 treatments x 2 biological replicates). From these, 283,586,752 reads were obtained from the susceptible line and 257,799,744 reads from the resistant line. Approximately 64% of the sequenced reads (346,953,900 mapped reads) were aligned to the *P*. *vulgaris* reference genome, and about 19,000 cowpea unigenes were identified from a total of 27,197 genes in *P*. *vulgaris*. An overview of the sequencing process is shown in S1 Table. The distribution of the mapped reads had similar patterns among the cDNA libraries.

### Analysis of differentially expressed genes

RNA-Seq was performed as an additional approach to identify candidate resistance genes in cowpea. The total number of transcripts was used for differential expression analysis using different combinations. The first analysis aimed at the characterization of differences in the basal level of expressed genes between the two NILs. For this comparison, the transcriptomes of the resistant (R NIL) and susceptible (S NIL) NILs were compared at 15 and 21 days after germination (corresponding to the RKN inoculated plants at 3 and 9 DAI, respectively). A total of 1,163 DEG were identified in mock-inoculated plants at both time points. From these, 548 and 556 genes were specifically differentially expressed by plants at the 15- and 21-day stages in resistant compared to susceptible plants, respectively, and 32 genes were differentially expressed at both time-points.

**Fig 5:**
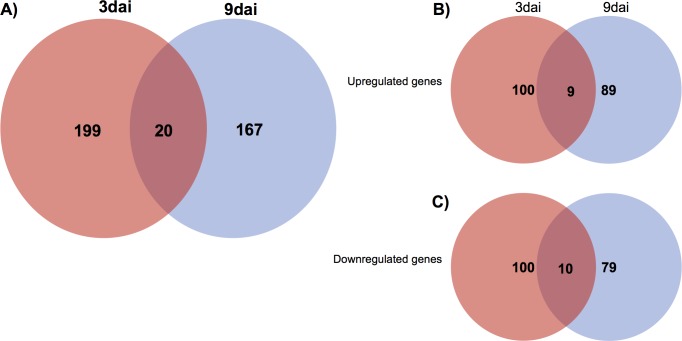
Differentially expressed genes (DEG) during RKN infection of near-isogenic lines of *Vigna unguiculata* roots. (A) Venn diagram showing DEG at 3 and 9 DAI of resistant NIL plants compared to susceptible NIL plants. The intersections represent genes commonly expressed between both time-points. (B) Venn diagram showing DEG up-regulated 3 and 9 DAI. The intersection represents genes commonly up-regulated. (C) Venn diagram showing DEG down-regulated 3 and 9 DAI. The intersection represents genes commonly up-regulated. FDR ≤ 0.1, fold change >± 1.

**Fig 6:**
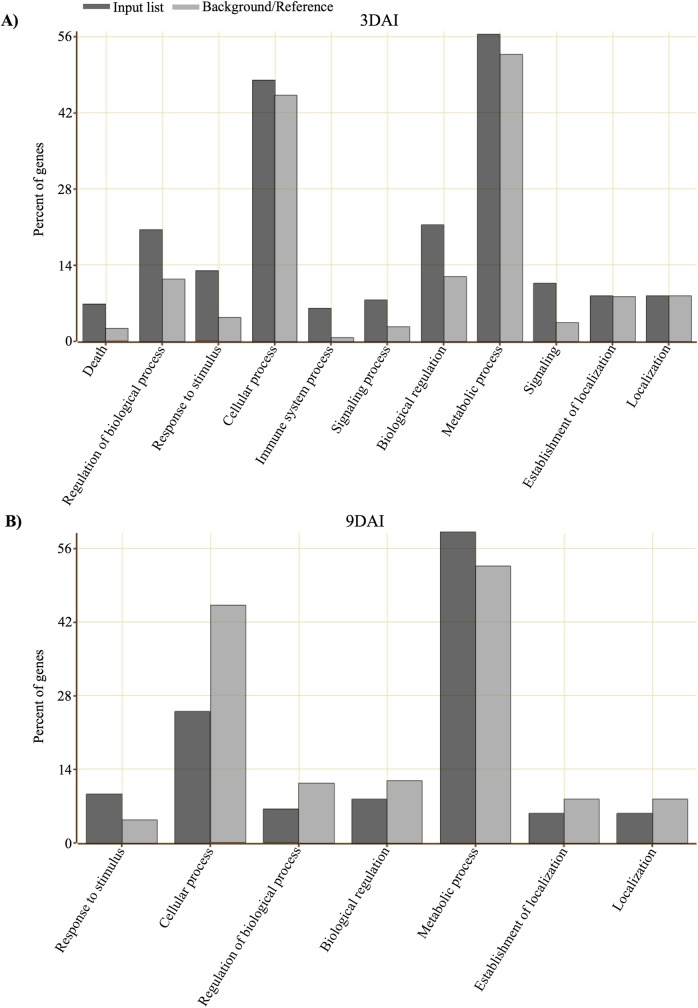
Gene ontology (GO) enrichment analysis of DEG 3 and 9 DAI of the resistant NIL compared to the susceptible NIL. **(**A) Number of DEG 3 DAI characterized according to biological process. (B) Number of DEG 9 DAI characterized according to biological process. GO based on *Phaseolus vulgaris* annotations.

Considering the high genome similarity between the two mock-inoculated NILs, the transcriptomes of the inoculated R NIL were compared to the transcriptomes of the inoculated S NIL at both 3 and 9 DAI. A total of 386 genes were significantly differentially expressed by the R NIL compared to the S NIL, at both time-points. Of these, 199 were specifically expressed at 3 DAI, 167 specifically expressed at 9 DAI and 20 genes were commonly regulated at both time-points (Fig 5A). Analysis of DEG showed slightly increased numbers of transcripts upregulated at 3 DAI as compared to 9 DAI (Fig 5B). The same trend was found for downregulated transcripts (Fig 5C). Gene ontology (GO) analysis was used to characterize the DEG and showed enrichment for terms involved in metabolic and cellular processes (Fig 6A and 6B).

The complete list of DEG in inoculated plants at 3 and 9 DAI were searched against the BAC sequences within SNP markers of both QTLs anchored after filtering by the best synteny with *P*. *vulgaris* chromosomes. Among the DEG, three and 12 genes were found within the *QRK-vu9*.*1* and *QRK-vu11*.*1* QTL, respectively. Of these 15 genes, six belong to the TIR-NBS-LRR family of resistance genes (Phvul.004G139900, Phvul.004G140400, Phvul.004G140500, Phvul.004G140700, Phvul.004G140800 and Phvul.011G030000). Notably, three genes (Phvul.004G140400, Phvul.004G140500 and Phvul.011G030000) were upregulated in at least one of the time-points investigated (Table 1).

**Table 1:** List of differentially expressed genes in RKN-inoculated cowpea plants, identified in BAC sequences [16] containing SNP markers for the *QRk-vu9*.*1* and *QRk-vu11*.*1* regions and filtered by the best synteny and blast hit between *Phaseolus vulgaris* and *Vigna unguiculata*.

ID^a^	Chr^b^	Annotation^c^	E-score	Exponent	Hit length	Cowpea LG
Phvul.004G039700	4	Malectin/receptor-like protein kinase family protein	0	999	2261	11
Phvul.004G039800	4	Malectin/receptor-like protein kinase family protein	0	999	2422	11
Phvul.004G040300	4	Malectin/receptor-like protein kinase family protein	0	999	1922	11
Phvul.004G055000	4	Plant protein of unknown function (DUF247)	0	999	761	11
Phvul.004G056400	4	Unknown function	0	999	906	11
Phvul.004G056900	4	Ribonuclease inhibitor	0	999	957	11
Phvul.004G059800	4	Unknown function	0	999	481	11
Phvul.004G139900	4	disease resistance protein (TIR-NBS-LRR class), putative	0	999	1134	11
Phvul.004G140400	4	disease resistance protein (TIR-NBS-LRR class), putative	0	999	1120	11
Phvul.004G140500	4	disease resistance protein (TIR-NBS-LRR class), putative	0	999	1107	11
Phvul.004G140700	4	disease resistance protein (TIR-NBS-LRR class), putative	1E-125	125	1117	11
Phvul.004G140800	4	disease resistance protein (TIR-NBS-LRR class), putative	0	999	1136	11
Phvul.011G001100	11	extra-large G-protein 1	0	999	721	9
Phvul.011G029900	11	Ankyrin repeat family protein	0	999	675	9
Phvul.011G030000	11	disease resistance protein (TIR-NBS-LRR class), putative	0	999	799	9

^a^The *Phaseolus vulgaris* gene annotation (Phytozome) ID.

^b^*P*. *vulgaris* chromosome position.

^c^Annotation of common bean homolog genes.

Interestingly, comparison between RKN-inoculated R NIL and mock-inoculated R NIL as well as comparison between RKN-inoculated S NIL and mock-inoculated S NIL at both time-points revealed 31 DEG that were localized in the BAC sequences within SNP markers of both QTLs anchored after filtering by the best synteny with *P*. *vulgaris* chromosomes. Notably, of these 31 genes, only 2 (Phvul.004G140700 and Phvul.004G140500) were also differentially regulated when the comparison was performed between inoculated R NIL and inoculated S NIL.

### Validation of RNA-Seq data by qRT-PCR

**Fig 7:**
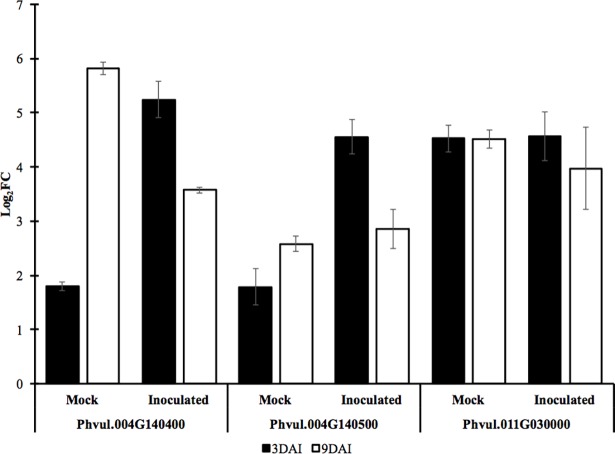
Differential expression of three RKN candidate genes in mock and inoculated cowpea plants 3 and 9 DAI. The expression was measured by qRT-PCR using cowpea Elongation factor gene as reference (Housekeeping gene) and the susceptible NIL as control.

To validate RNA-Seq analysis results and to investigate the expression of selected DEG localized in the QTL regions, the expression of eight genes was further characterized using qRT-PCR in roots inoculated with RKN or mock-inoculated at two time-points (3 and 9 DAI), and specific primers designed based on cowpea BAC sequences (S2 Table). The qRT-PCR analysis demonstrated that, overall, the gene expression quantified was positively correlated with RNA-Seq expression pattern (Table 2 and S3 Table). Analysis of the comparison between R NIL and S NIL transcripts revealed three R gene-like transcripts with homology to Phvul.004G140400, Phvul.004G140500 and Phvul.011G030000 that were upregulated at both time-points (3 and 9 DAI), independent of RKN presence, with a reduction in the expression at 9 DAI compared to 3 DAI (Table 2 and Fig 7).

**Table 2:** Fold change (Log_2_FC) of eight differentially expressed genes using qRT-PCR for RNA-Seq validation using the susceptible near-isogenic line as control.

	Comparison							
Gene ID	Rk-NI-3 x Null-NI-3^a^		Rk-NI-9 x Null-NI-9^a^		Rk-IN-3 x Null-IN-3^b^		Rk-IN-9 x Null-IN-9^b^	
	(RNA-seq)^c^	(qRT-PCR)^d^	(RNA-seq)^c^	(qRT-PCR)^d^	(RNA-seq)^c^	(qRT-PCR)^d^	(RNA-seq)^c^	(qRT-PCR)^d^
Phvul.004G140400	2.37	1.80	3.40	5.82	5.02	5.24	3.37	3.57
Phvul.004G140500	1.31	1.64	1.36	2.50	1.46	4.55	0.67	2.86
Phvul.004G140700	-2.95	-4.96	-2.81	-4.63	-3.93	-3.74	-0.81	-4.41
Phvul.004G140800	-	-	-	-	-1.44	-	-	-
Phvul.011G030000	5.00	4.53	4.24	4.51	4.74	4.55	-	3.43
Phvul.011G181500	-	-	-	-	-2.79	-	-	-
Phvul.011G033900	-	-	0.82	0.56	1.22	-	1.33	-
Phvul.011G029900	0.82	-	0.98	-0.22	0.79	-	1.02	-

^a^Mock-inoculated.

^b^RKN inoculated.

^c^FDR ≤ 0.1.

^d^*p* ≥ 0.05 (T-test).

When mock-inoculated plants were used as control, the gene expression difference of these three genes (Phvul.004G140400, Phvul.004G140500 and Phvul.011G030000) was not detected using qRT-PCR, confirming the preexisting difference of basal expression levels between NILs rather than response to RKN inoculation. In fact, comparison between inoculated and mock-inoculated NIL allowed detection of differential expression of only two genes analyzed (Phvul.004G140700, Phvul.011G033900), from which only Phvul.004G140700 was localized under the *QRk-vu9*.*1* region (S3 Table).

### Characterization of candidate genes using the *V*. *unguiculata* gene expression atlas

The cowpea gene homolog to Phvul.004G140400 in common bean showed similarity with 59 cowpea transcript sequences available in the *Vigna unguiculata* gene expression atlas [27]. Two of these transcripts, Vun_T16420.1 (99% coverage and 87% identity to Phvul.004G140400) and Vun_T16418.1 (87% coverage and 84% identity to Phvul.004G140400) presented a high bit score with 1312 and 573 bp, respectively. Vun_T6420.1 sequence encodes a NB-ARC domain with similarity to XP_017439631.1 (PREDICTED: TMV resistance protein N-like X3 [*Vigna angularis*]), XP_017439629.1 (PREDICTED: TMV resistance protein N-like X1 [*Vigna angularis*]), XP_017439630.1 (PREDICTED: TMV resistance protein N-like X2 [*Vigna angularis*]) (99% coverage and 90% identity). Vun_T16418.1 encodes a TIR domain, also with similarity to the cowpea gene homolog to Phvul.004G140400. Based on expectation (e-value) threshold, 70 transcripts had similarity to Phvul.004G140500, with Vun_T16712.2 and Vun_T22052.9 showing higher bit scores, 585 and 400 bp. Vun_T16712.2 sequence with a TIR domain was better aligned to BAU02586.1 (Hypothetical protein VIGAN_11213900 [*Vigna angularis* var. *angularis*]) with 99% coverage and 82% identity. Interestingly Vun_T22052.9, another transcript with a TIR domain, had better alignment with a *Phaseolus vulgaris* XP_007152563.1 (Hypothetical protein PHAVU_004G140700g [*Phaseolus vulgaris*]), but not with PHAVU_004G140500g. The gene Phvul.011G030000 had similarity to two transcripts in the cowpea transcriptome with bit scores of 54 bp. Both transcripts, Vun_T16420.4 and Vun_T16420.3, encode a NB-ARC domain and have high similarity to XP_014501488.1 (PREDICTED: TMV resistance protein N-like isoform X2 [*Vigna radiata* var. *radiata*]) and XP_014501487.1 PREDICTED: TMV resistance protein N-like isoform X1 [*Vigna radiata* var. *radiata*]). All these genes are mainly expressed in root tissues (S2 Fig). Apparently, all six transcripts (Vun_T16420.1, Vun_T16418.1, Vun_T16420.3, Vun_T16712.2, Vun_T22052.9 and Vun_T16420.4) are mainly co-expressed with other genes, also transcripts in the roots according to the cowpea gene expression atlas. Among the co-expressed transcripts, genes such as Disease resistance protein family (TIR-NBS-LRR class), WRKY family transcription factor, Leucine-rich repeat protein kinase family (LRR-RK), Auxin-responsive family protein and Plant invertase / pectin methylesterase inhibitor superfamily were identified as having 99% correlation and high expression. A list with co-expressed transcripts can be found in S4 Table.

### Protein domain characterization from candidate genes

Because the three cowpea genes, homologs to Phvul.004G140400, Phvul.004G140500 and Phvul.011G030000, localized in the investigated QTLs have constitutively higher expression in resistant plants than in susceptible plants, the proteins encoded by these genes were characterized further. The proteins encoded by the 3 genes are 621, 960 and 667 amino acids in length, respectively, and have amino acid identity ranging from 44 to 58% among them (Table 3).

**Table 3:** Percent identity matrix among cowpea predicted proteins homologous to common bean Phvul.011G300000, Phvul.004G140400 and Phvul.004G140500 proteins.

Protein	Phvul.011G300000_homolog	Phvul.004G140400_homolog	Phvul.004G140500_homolog
**Phvul.011G300000_homolog **	100%		
**Phvul.004G140400_homolog**	44%	100%	
**Phvul.004G140500_homolog**	46%	58%	100%

**Fig 8:**
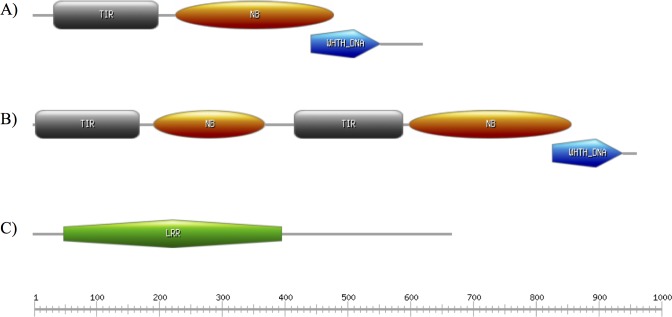
Conserved domains of three cowpea predicted proteins encoded by candidate genes constitutively higher expressed in RKN resistant plants. Protein encoded by the cowpea gene homolog of (A) Phvul.004G140400 in common bean, (B) Phvul.004G140500 and (C) Phvul.011G300000. The scale bar represents the number of amino acids.

The gene homolog to Phvul.004G140400 in cowpea encodes a protein with TIR (Toll/interleukin-1 receptor homology) and NB-ARC (Nucleotide-binding) domains, commonly found in resistance genes. Additionally, the C terminal region of this protein has a Winged HTH binding domain (winged helix-turn-helix DNA-binding domain) (Fig 8A). The cowpea gene homolog of Phvul.004G140500 encodes a protein that seems to have two TIR-NBS proteins fused together, in the following sequence: TIR and NB-ARC domains followed by TIR, NB-ARC and Winged HTH binding domain (Fig 8B). Finally, the gene homolog to Phvul.011G300000 encodes a protein with a leucine-rich repeat domain (Fig 8C). The first two proteins are predicted to be cytoplasm localized, while the last protein is predicted to have nuclear localization, according to amino acid sequence analysis performed using CELLO2GO [31].

To characterize the relationship among the candidate genes and between these genes and others with high amino acid similarity, we built a phylogenetic tree using the complete sequence of amino acids of three predicted cowpea proteins and 11 proteins sharing similarity to each of them. As expected, the phylogenetic tree separated the three proteins into different clades, grouping each of them with their 11 related proteins. All three proteins showed high similarity with their respective homologues in *P*. *vulgaris* and *Vigna* species (S3 Fig).

## Discussion

Plant genetic resistance is the most sustainable way to reduce yield loss caused by RKN in many crops, including cowpea. Mapping QTLs and finding candidates genes responsible for nematode resistance provides a valuable tool for breeding programs through the identification of genetic markers for effective marker-assisted selection, thus enabling efficient incorporation of desirable traits into improved cowpea cultivars. In this study, we identified a major effect QTL on cowpea LG 9 associated with RKN resistance. The skewed or bi-modal phenotypic distribution observed for the number of egg masses per root system trait (Fig 4) supports the current mapping of the *QTL QRk-vu9*.*1*. The RKN resistance QTL mapped in this study and the previously mapped *QRk-vu11*.*1* were confirmed by genotyping results of susceptible and resistant NILs derived from conventional backcrossing. The QTL *QRk-vu9*.*1* position does not correspond to the *Rk*^*2*^ locus described by Roberts et al. [11]. In that earlier study, the authors examined the inheritance of the resistance in IT84S-2049 against two populations of *M*. *incognita* (virulent and avirulent to gene *Rk*) and concluded that the high resistance in IT84S-2049 was associated with the *Rk* gene locus, which was hypothesized to be a complex RKN resistance locus harboring both the *Rk* and the *Rk*^*2*^ genes in a tightly linked manner, because of the lack of any susceptible recombinants among a total of 1206 F2 and 1144 TC1 plants tested. The fact that the *QTL QRk-vu9*.*1* was not mapped to the region of *Rk* locus indicates that the locus conferring the resistance to *M*. *javanica* in the current study is different from *Rk*^*2*^. Possibly, this new locus, when combined with the *Rk* gene or its variants, also confers some resistance to virulent populations of *M*. *incognita*. The present work showed that the NILs containing favorable alleles only in *QRk-vu9*.*1* had a weak resistance response to *M*. *javanica*. However, when *QRk-vu9*.*1* was combined with positive alleles at *QRk-vu11*.*1*, the plants displayed a much stronger resistance response (Fig 3). Previous investigations showed that *Rk*-mediated resistance did not suppress root penetration by infective juveniles of *M*. *incognita*, but the nematodes that established feeding sites in resistant roots exhibited arrested development by 14 DAI [35]. Interestingly, our results showed that the resistance controlled by the combination of both *QRk-vu9*.*1* and *QRk-vu11*.*1* had an additive effect, interfering with *M*. *incognita* development in cowpea roots in early stages of feeding site establishment (3 DAI).

The candidate gene discovery strategy taking advantage of the synteny between cowpea and common bean allowed for the identification of candidate resistance genes, encoding proteins such as TIR-NBS-LRR, LRR Kinase, NB-ARC domain-containing disease resistance protein (S4 File), all localized in the common bean syntenic regions of *QRk-vu9*.*1* and *QRk-vu11*.*1*. Fifteen of these genes were also differentially expressed between resistant and susceptible plants with three of these genes (homologous to TIR-NBS-LRR genes of *Phaseolus vulgaris*) showing a higher expression in the resistant plant. Currently, most of the cloned nematode R-genes encode proteins belonging to the largest class of R-proteins with central nucleotide-binding (NB) and C-terminal leucine-rich repeat (LRR) domains. These R-genes include *Mi-1*.*2*, *Mi-9*, *Hero*, *Gpa2* and *Gro1-4* [36–40]. Most of these genes encode N-terminal coiled-coil (CC) domain-containing NB-LRR proteins, with the exception of *Gro1-4*, which encodes a N- terminal Toll-interleukin 1 receptor (TIR) NB-LRR protein. Additional characterized R-genes with distinct domain conformation include *Hs1*^*pro*-1^ from sugar beet and *Rhg1* and *Rhg4* from soybean. *Hs1*^*pro*-1^, the first cloned nematode R-gene, encodes a leucine-rich protein with a transmembrane domain, which has little similarity to other R-proteins, while *Rhg1* and *Rhg4* encode proteins with extracellular LRRs, a transmembrane domain and a cytosolic serine-threonine kinase domain [41–43]. Notably, these resistance genes are constitutively expressed throughout plant tissues and their expression does not respond to nematode inoculation [38, 39, 44]. Consistent with the constitutive expression of R genes, the best candidate genes (Phvul.004G140400, Phvul.004G140500 and Phvul.011G030000) evaluated in the present investigation did not respond to RKN inoculation when compared to mock-inoculated control plants. Nevertheless, the basal level of gene expression in resistant plants was significantly elevated in comparison to susceptible plants, suggesting the resistant plants may display increased sensitivity to RKN detection.

The three best candidate genes with differential expression, besides having a high similarity with their homologs in *Phaseolus vulgaris*, also have high homology with TMV resistance protein N-like genes of *Vigna radiata*. The *N* gene is a member of the Toll-interleukin-1 receptor/nucleotide-binding site/leucine-rich repeat (TIR-NBS-LRR) class of plant resistance (*R*) genes and confers resistance to *Tobacco mosaic virus* (TMV) in tobacco plants [45]. Homologous genes of the *N* gene have been identified in other species of plants conferring resistance to diseases such as potato resistance to *Synchytrium endobioticum* [46].

It is noteworthy that the candidate genes selected in this work are mainly expressed in roots [27]. Using the same source of information, we found other genes such as Disease resistance protein (TIR-NBS-LRR class) family, WRKY family transcription factor (known to interact with NBS-LRR proteins [47]) and Leucine-rich repeat protein kinase family protein to be co-expressed with transcripts showing high similarity with our candidate genes.

The proteins encoded by the homologous genes Phvul.004G140400 and Phvul.004G140500 contain TIR and NB-ARC domains, with Phvul.004G140500 encoding these domains in duplicate. Interestingly, these genes do not seem to encode a protein containing a LRR domain. In the case of Phvul.011G030000 only the LRR domain was identified. The LRR domains are the most polymorphic part of the plant NBS-LRR proteins, which likely reflects their role in effector recognition. In addition to its role in effector recognition, the LRR domain also plays an important role in keeping NBS-LRR proteins in the “off” state. Studies have demonstrated that the LRR domain physically associates with the NB-ARC domain [48, 49] and deletion of the LRR domain typically results in auto-activation [49, 50]. The absence of a LRR domain could justify the higher basal expression of the first two genes as detected in our results.

Future functional analysis of cowpea genes homologous to Phvul.004G140400, Phvul.004G140500 and Phvul.011G030000 will enhance our understanding of *Rk*-mediated RKN resistance and provide ‘perfect markers’ which would further improve marker-assisted breeding efficiency. In addition, this would determine exactly the gene responsible for RKN resistance under each QTL, allowing insertion of these genes through genetic transformation into other legume crop plants of major economic importance, such as common bean and soybean.

## Conclusions

In the present study, a major QTL (*QRk-vu9*.*1*) associated with RKN resistance was mapped on cowpea LG 9 associated with RKN resistance. This QTL does not correspond to the *Rk*^*2*^ locus which was previously found to be associated with strong RKN resistance linked to the *Rk* gene region, and therefore represents a novel RKN resistance locus. Possibly, this new locus, when combined with the *Rk* gene or its variants, confers some resistance to virulent populations of *M*. *incognita*. We showed that the NILs containing favorable alleles only in *QRk-vu9*.*1* responded with a weak resistance to *M*. *javanica*. However, when *QRk-vu9*.*1* was combined with positive alleles at *QRk-vu11*.*1*, plants displayed a much stronger resistance phenotype. Based on RNA-seq data and the synteny between *V*. *unguiculata* and *P*. *vulgaris*, we were able to select three promising genes (homologous to Phvul.004G140400, Phvul.004G140500 and Phvul.011G030000) encoding proteins belonging to the class of R-proteins with central nucleotide-binding (NB) and C-terminal leucine-rich repeat (LRR) domains, localized in the regions of *QRk-vu9*.*1* and *QRk-vu11*.*1*. These three best candidate genes with high similarity to their homologs in *Phaseolus vulgaris*, also had high homology with TMV resistance protein N-like genes of *Vigna radiata*. Functional analysis of these genes will provide best markers to be used in marker-assisted breeding and will allow the transfer of these genes to other legume crops through genetic transformation.

## Supporting information

S1 Fig Genetic map derived from the 524B x IT84S-2049 recombinant inbred line (RIL) population. The 11 linkage groups were named and oriented according the cowpea consensus map [16]. Each marker is represented with a horizontal line.
(TIFF)

S2 Fig Gene expression of six cowpea transcripts with similarity to Phvul.004G140400, Phvul.004G14500 and Phvul.011G030000 in different tissues of plant. (TIFF)

S3 Fig Phylogenetic tree of *Vigna unguiculata* proteins and 33 proteins with similarity available in NCBI data bank using a Neighbor-Joining method. The distances were computed using the Poisson correction method and are in the units of the number of amino acid substitutions per site. All ambiguous positions were removed for each sequence pair. There was a total of 1968 positions in the final dataset. The predicted cowpea proteins are highlighted in yellow. A *Prunus persica* protein was used as an outgroup.
(TIFF)

S1 Table An overview of RNA-seq reads alignment stats of cowpea, CB46 72-1-3 (resistant) and CB46 Null (susceptible), inoculated and mock inoculated with RKN mapped in *Phaseolus vulgaris* reference genome. *NI = Mock inoculated treatment; IN = Inoculated treatment; 3 = three days after inoculation; 9 = nine days after inoculation.
(XLSX)

S2 Table Primer sequences used to validade the RNA-seq with qRT-PCR. *Primers amplify 200 bp to DNA template.
(XLSX)

S3 Table Fold change (Log2FC) of eight differentially expressed genes using qRT-PCR for RNA-seq validation using non-inoculated NIL as control. ^1^ FDR ≤ 0.1; ^2^ p ≥ 0.05 (T-test).
(XLSX)

S4 Table Co-expressed transcripts correlated with other transcripts aligned with three resistance candidate genes. *Black-eyed pea Gene Expression atlas (http://vugea.noble.org) [27].
(XLSX)

S1 File The co-location of the major QTL *QRk-vu9*.*1* and the donor region of the newly mapped gene-type resistance in near-isogenic lines derived from conventional backcrossing. (XLSX)

S2 File The co-location of the major QTL *QRk-vu11*.*1* and the loss of *Rk* region in susceptible near-isogenic lines derived from conventional backcrossing. (XLSX)

S3 File Genetic comparison between CB46 72-1-3(6) (resistant) and CB46 Null(5) (susceptible) cowpea NILs based on marker SNPs. (XLSX)

S4 File List of genes found into cowpea BAC sequences anchoring SNP markers in the *QRk-vu9*.*1* and *QRk-vu11*.*1* region based in the best synteny between cowpea and common bean. (XLSX)
