# Supplementary figures and images for: QTL mapping and transcriptome analysis of cowpea reveals candidate genes for root-knot nematode resistance
Source: PLoS One. 2018 Jan 4;13(1):e0189185. doi: 10.1371/journal.pone.0189185 (PMC5754085; doi:10.1371/journal.pone.0189185)

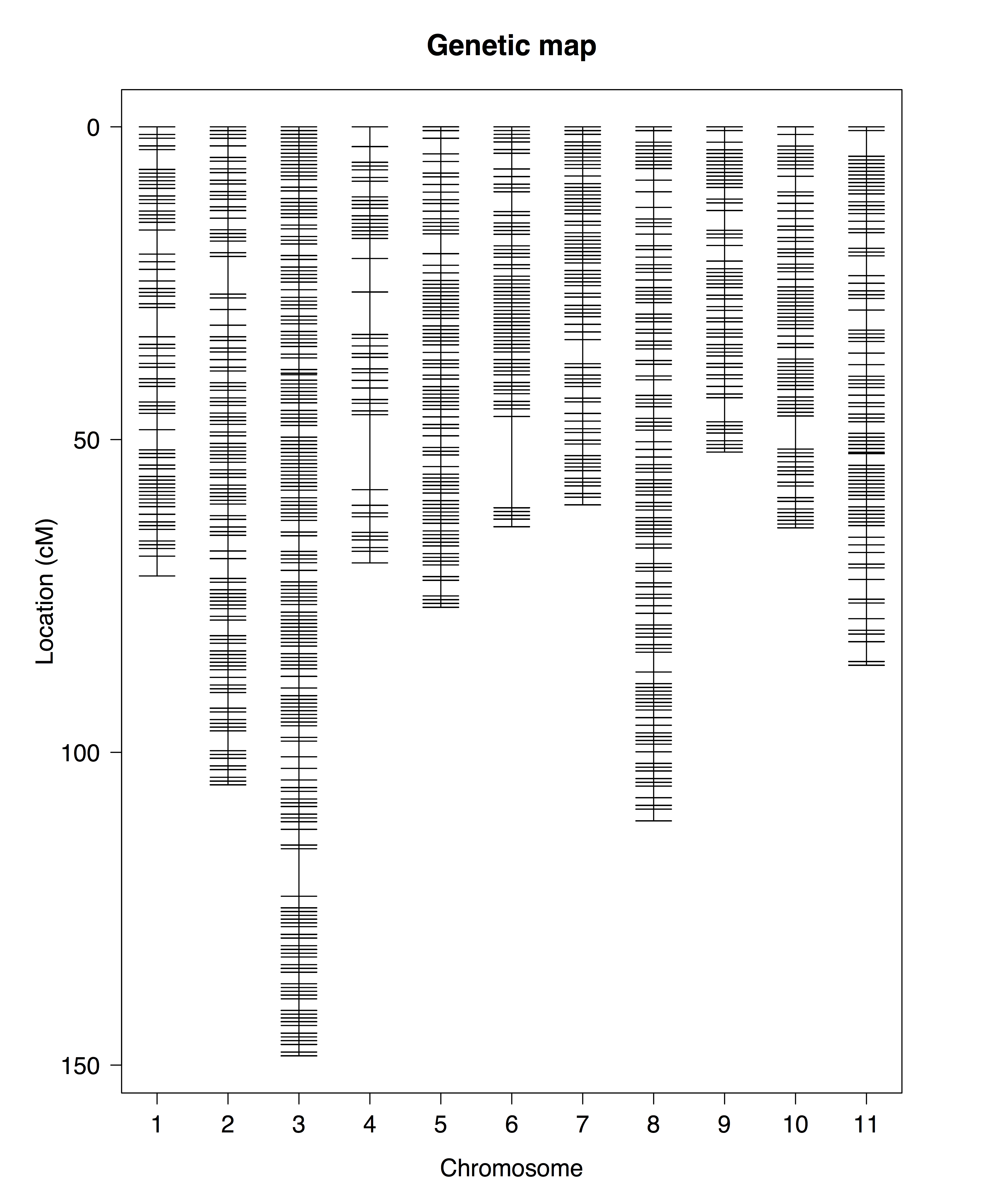

Supplement: S1 Fig — The 11 linkage groups were named and oriented according the cowpea consensus map [16]. Each marker is represented with a horizontal line. (TIFF) [file pone.0189185.s001.tiff]

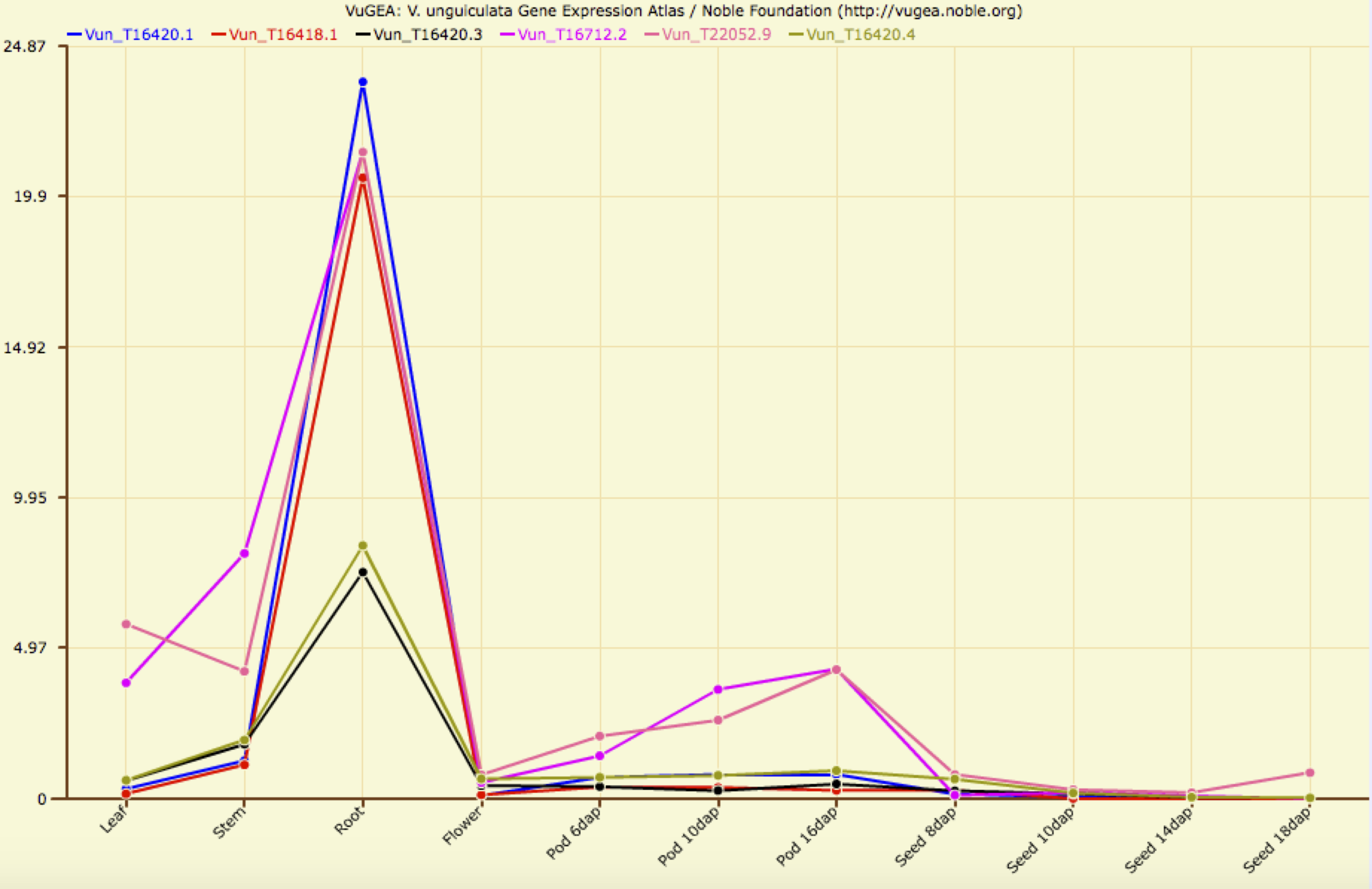

Supplement: S2 Fig — (TIFF) [file pone.0189185.s002.tiff]

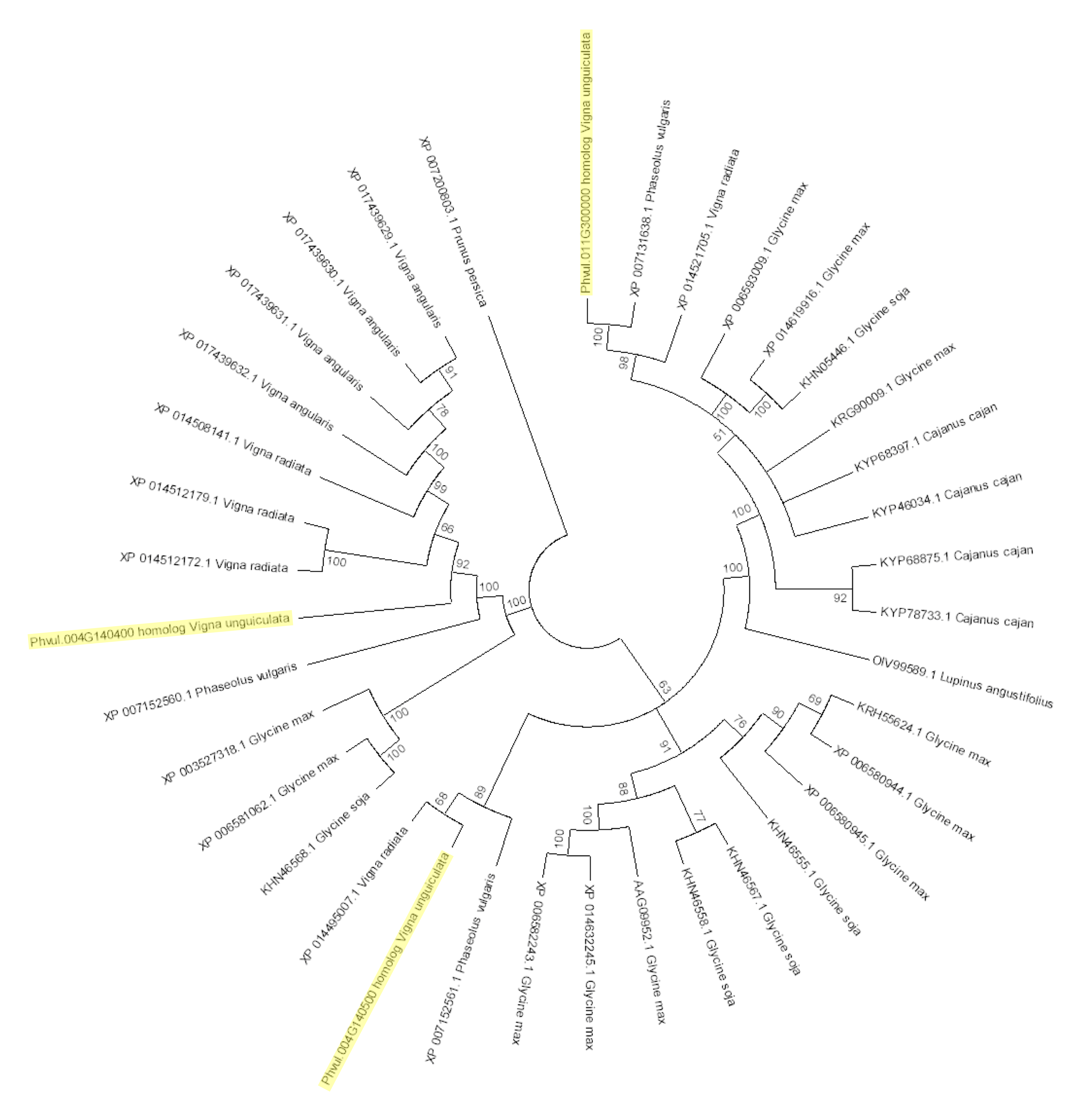

Supplement: S3 Fig — The distances were computed using the Poisson correction method and are in the units of the number of amino acid substitutions per site. All ambiguous positions were removed for each sequence pair. There was a total of 1968 positions in the final dataset. The predicted cowpea proteins are highlighted in yellow. A Prunus persica protein was used as an outgroup. (TIFF) [file pone.0189185.s003.tiff]
